# Quinine and the Hearing

**Published:** 1885-02

**Authors:** 


### QUININE AND THE HEARING.

It may not be generally known that the free use of the common remedy, quinine, is attended with danger to the ear. Dr. Greene has recently published a paper on the subject in a medical journal. His conclusions are:

1. Clinical experience the world over is, that *quinine* occasionally produces serious injury to the ears. 2. From our present knowledge, both clinical and experimental, we are justified in asserting that the action of *quinine* upon the ears is to produce congestion of the labyrinth and tympanum and sometimes distinct inflammation, with permanent tissue-changes. 3. The action of the drug upon the ears should always be considered in prescribing it, and changes in the ears due to existing or previous inflammation of those organs, constitute a contra-indication to the medicine in large doses or for a long time, except under urgent circumstances. 4. Where large and continuous doses are absolutely necessary, an occasional intermission of the administration is desirable, if possible, to diminish the risk to the ears.

